# Supplementary material for: Health professionals’ knowledge on vaccine cold chain management and associated factors in Ethiopia: Systematic review and meta-analysis
Source: PLoS One. 2023 Nov 29;18(11):e0293122. doi: 10.1371/journal.pone.0293122 (PMC10686474; doi:10.1371/journal.pone.0293122)
Supplement: S1 File — (DOCX) [file pone.0293122.s002.docx]

**Newcastle Ottawa quality assessment of prevalence studies**

| **Included Studies** | **Assessment criteria** | | | | | | | |  |
| --- | --- | --- | --- | --- | --- | --- | --- | --- | --- |
|  | Representativeness of the sample | Sample size: | Non-respondents | Ascertainment of the exposure (risk factor): | The subjects in different outcome groups are comparable, based on the study design or analysis. Confounding factors are controlled | Assessment of the outcome: | Statistical test | total | Remark |
| Asres M et al | 1 | 1 | 1 | 1 | 1 | 2 | 1 | 8 | good |
| Degavi G et al | 1 | 1 | 1 | 2 | 1 | 2 | 1 | 9 | good |
| Feyisa D et al | 1 | 1 | 1 | 1 | 1 | 2 | 1 | 8 | good |
| Mohammed SA et al | 1 | 1 | 1 | 2 | 1 | 2 | 1 | 9 | good |
| Rogie B et al (unpublished) | 1 | 1 | 1 | 1 | 1 | 2 | 1 | 8 | good |
| Woldemichael B et al | 1 | 1 | 1 | 1 | 1 | 2 | 1 | 8 | good |
| Yassin ZJ et al | 1 | 1 | 1 | 2 | 1 | 2 | 1 | 9 | good |
| Erassa TE et al | 1 | 1 | 1 | 2 | 1 | 2 | 1 | 9 | good |
| Zelalem E et al(unpublished) | 1 | 1 | 1 | 2 | 1 | 2 | 1 | 9 | good |
