# Supplementary material for: Health professionals’ knowledge on vaccine cold chain management and associated factors in Ethiopia: Systematic review and meta-analysis
Source: PLoS One. 2023 Nov 29;18(11):e0293122. doi: 10.1371/journal.pone.0293122 (PMC10686474; doi:10.1371/journal.pone.0293122)
Supplement: Attachment — Submitted filename: Review.docx [file pone.0293122.s005.docx]

Dear authors thanks for this interesting review. In general, this review is well written. However, I have some comments and questions

1. Please incorporate your review Prospero registration number in abstract section
2. Please put the citation for the PRISMA guideline you used under method section. Is that PRISMA guideline 2020 or not?
3. If you used the PRSMA guideline 2020, some components were missed for example Certainty assessment
4. Please avoid the term data analysis and process in method section rather use Synthesis methods and Reporting bias assessment
5. Avoid a paragraph ‘’Fixed effect model would have been used 187 if studies were homogeneous’’
6. What were the limitations of your review?
7. Lastly, please use PRISMA 2020 <https://pubmed.ncbi.nlm.nih.gov/33782057/> guideline and rewrite your review and meta-analysis

Thank you
