# Supplementary material for: Health professionals’ knowledge on vaccine cold chain management and associated factors in Ethiopia: Systematic review and meta-analysis
Source: PLoS One. 2023 Nov 29;18(11):e0293122. doi: 10.1371/journal.pone.0293122 (PMC10686474; doi:10.1371/journal.pone.0293122)
Supplement: Attachment — Submitted filename: Reviewer comment.docx [file pone.0293122.s006.docx]

Reviewer (Mistire Teshome) comments on ‘**Manuscript Number:** PONE-D-23-03134
**Manuscript Title:** Health professionals’ Knowledge on Vaccine cold chain management and associated factors in Ethiopia: systematic review and meta-analysis’

***Major comments***

1. I have seen self-plagiarism from your previous published work (Kasahun AW, Zewdie A, Shitu S, Alemayehu G. Vaccine cold chain management practice and associated factors among health professionals in Ethiopia: systematic review and meta-analysis. Journal of Pharmaceutical Policy and Practice. 2023 Dec;16(1):1-0.).
2. You used a mnemonic PICO (population, intervention, control, outcome), we use it for effectiveness studies to develop question, instead of using CoCoPop (condition, context, population) for prevalence studies. I have doubt on your inclusion criteria since you use PICO. I need to see your search strategies and search terms that you use for each database.

***Minor comments***

1. On methods part line 118 needs citation.
2. You use Joanna Briggs Institute for data extraction, why not for quality assessment and analysis too?
